# Magnetic Anisotropy and Spin Coupling in a Cobalt(II) Dimer with Bioinspired Bridges

**DOI:** 10.1021/acsphyschemau.5c00134

**Published:** 2026-02-26

**Authors:** Alan Almeida, Ana Clara das Neves, Paula Brandão, Mariem Masmoudi, Luis Ghivelder, Clebson Cruz, Mario Reis

**Affiliations:** † Instituto de Física Armando Dias Tavares, 28130 Universidade do Estado do Rio de Janeiro, R. São Francisco Xavier 524, Rio de Janeiro 20550-900, RJ, Brazil; ‡ Centro de Ciências Naturais e Humanas, 425753 Universidade Federal do ABC, Santo André 09210-580, SP, Brazil; § Department of Chemistry, CICECO, 56062 University of Aveiro, 3810-193 Aveiro, Portugal; ∥ Instituto de Física, Universidade Federal do Rio de Janeiro, Av. Athos da Silveira Ramos 149, Rio de Janeiro 21941-909, RJ, Brazil; ⊥ Centro das Ciências Exatas e das Tecnologias, 423876 Universidade Federal do Oeste da Bahia, Rua Bertioga 892, Morada Nobre, Barreiras 47810-059, BA, Brazil; # Instituto de Física, 28110 Universidade Federal Fluminense, Av. Gal. Milton Tavares de Souza s/n, Niteroi 24210-346, RJ, Brazil

**Keywords:** hexacoordinated Co(II), molecular magnetism, zero-field splitting, magnetic anisotropy, adenine bridging

## Abstract

Cobalt­(II) metal complexes constitute a versatile platform for investigating how coordination geometry and spin–orbit coupling determine their magnetic properties. Although numerous cobalt­(II) coordination complexes have been reported in recent literature, only a limited number exhibit comprehensive and quantitatively reliable magnetic characterization. In this work, we investigate the magnetic properties of the hexacoordinated cobalt dimer [Co_2_(μ-L1H)_2_(μ-H_2_O)_2_(H_2_O)_4_]­4NO_3_·2H_2_O, where L1H denotes the adenine bridging ligand. The hexacoordinated environment stabilizes a high-spin *S* = 3/2 configuration for both Co­(II) centers, resulting in strong spin–orbit coupling and significant zero-field splitting, described by axial (*D*) and rhombic (*E*) anisotropy parameters. Fits to magnetic susceptibility and magnetization data reveal antiferromagnetic coupling between the Co­(II) ions, with a ratio of *E*/*D* ≈ 1/4 and *D*/*k*_B_ = 89 K, evidencing pronounced magnetic anisotropy in the system. This behavior is further supported by anisotropic Landé factors *g*_*x*_ = *g*_*y*_ = 2.5 and *g*_*z*_ = 2.4, consistent with easy-plane magnetic anisotropy.

## Graphical abstract

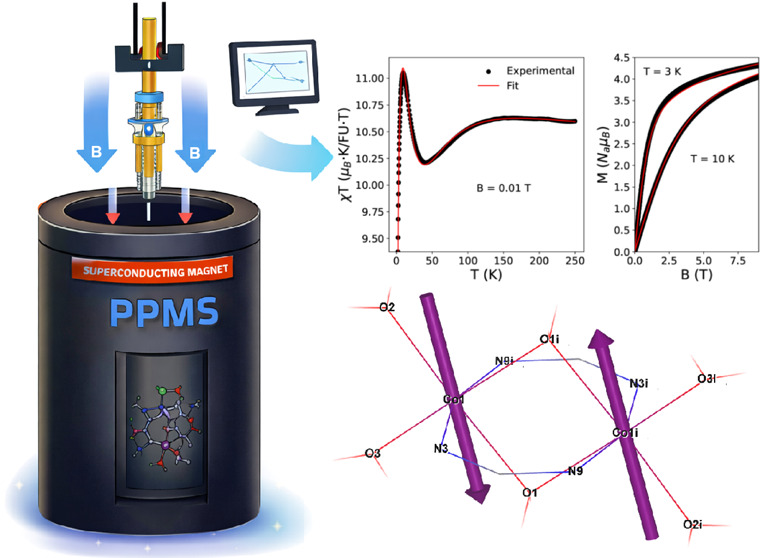

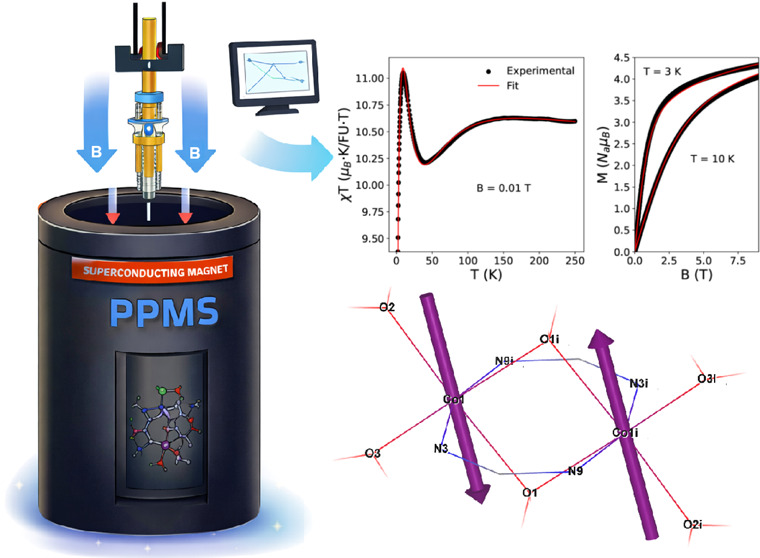

## 1 Introduction

The study of metal complexes lays the groundwork for a broad spectrum of technologies, from magnetic refrigeration,^1^ high-density magnetic recording,^2^ and spintronics^3^ to catalysis^4,5^ and biomedical applications.^6^ Certain metal–organic compounds also exhibit noteworthy cytotoxic activity^7–10^ and have begun to feature in emerging quantum devices, such as quantum batteries and quantum thermodynamic devices.^11,12^ Within this broad landscape of metal–organic systems, dinuclear transition-metal complexes occupy a particularly relevant position, as they provide well-defined magnetic units in which exchange interactions and anisotropy effects can be quantitatively assessed.^1,13,14^ In this context, adenine-based coordination compounds offer structurally robust frameworks capable of stabilizing closely spaced metal centers, making them suitable model systems for detailed magnetochemical investigations.^6,15^

At the same time, although numerous Co­(II) complexes have been reported, only a limited number of these exhibited a complete and consistent determination of magnetic anisotropy parameters, as supported by both magnetometry and spectroscopy data. This limited availability of well-characterized systems hampers a deeper understanding of how spin–orbit coupling rules the magnetic behavior of such systems.

From this perspective, the present work examines the dinuclear cobalt complex [Co_2_(μ-L1H)_2_(μ-H_2_O)_2_(H_2_O)_4_]­4NO_3_·2H_2_O (compound **I**), where L1H = adenine. The dinuclear cobalt complex investigated here was originally synthesized and structurally characterized by Masmoudi et al.^15^ in the context of cytotoxic studies involving adenine-based coordination compounds. While that work focused primarily on the biological activity of the system, the magnetic properties of this compound remained unexplored. In this study, we shift the emphasis to a detailed magnetic characterization of compound **I**, aiming to elucidate its anisotropy and magnetic coupling mechanisms. Such an analysis contributes to the growing body of studies on molecular magnetic systems, for which precise and quantitative magnetic parameters are essential to assess their physical behavior and potential relevance in broader condensed-matter and quantum-magnetism contexts.^11,12,16,17^

In this regard, by providing a comprehensive experimental–theoretical characterization of compound **I**, our work offers valuable reference data for ongoing efforts to correlate structure, anisotropy, and function in Co-metal complexes. Following a description of its crystal structure, we propose an effective magnetic Hamiltonian, present magnetization and χ*T* measurements, and analyze the model against both data. The extracted parameters give quantitative information on exchange interactions, anisotropy, and the mechanisms that govern the magnetic response of the complex, and the comprehensive magnetic characterization reported provides consistent parameters for comparison of future studies on anisotropy-driven phenomena in cobalt­(II) molecular systems, highlighting the role of anisotropy in hexacoordinated compounds.

## 2 Experimental Section

### 2.1 Synthesis

The dinuclear cobalt­(II) was synthesized by the reaction, in 15 mL of ethanol, of cobalt nitrate hexahydrate (1.0 mmol, 0.291 g) and adenine (1.0 mmol, 0.135 g) under continuous stirring conditions at room temperature for 2 h. Pink-plate-like crystals suitable for single X-ray analysis were obtained by slow evaporation of the resultant solution. Elemental analysis (%): Calcd. (based on single-crystal data) for C_10_H_26_N_14_O_20_Co_2_: C, 15.4; H, 3.3; N, 25.1; found: C, 15.9; H, 3.5; N, 25.5.

The IR spectrum shows the characteristic bands of adenine. The *n*(NH_2_) appears at 3115 cm^–1^, and the *n*(NH_2_) is found at 1510 cm^–1^. The *n*(C=C) and *n*(C=N) vibrations of the heterocyclic ring are observed as a broad band at 1566 and 1677 cm^–1^, respectively. With respect to the vibrations of the nitrate group, the typical band appears at 1387 cm^–1^. The weak peaks at 544 and 551 cm^–1^ could be associated with metal–nitrogen and metal–oxygen vibrations.

### 2.2 Crystal Structure

The crystal structure of compound **I** (Figure 1) is described in detail in ref 15 by Masmoudi et al., where an isostructural analogue in which cobalt is replaced by copper is also reported. Here, we present a brief structural description that is relevant for magnetic interpretation. Compound **I** crystallizes in the monoclinic system with the space group *P*2_1_/*c* and features a dinuclear cobalt unit, in which two equivalent Co ions are bridged by two μ-adenine ligands and two μ-H_2_O molecules (Table 1). Each cobalt center is coordinated by two nitrogen atoms from the adenine ligands and four water molecules, completing a slightly distorted hexacoordinate environment. The structure exhibits a short Co–Co distance of 3.1241(6) Å (Figure 2). Relevant bond distances for compound **I** are given in Table 2. Charge-balance considerations, together with four nitrate counterions, indicate a +2 oxidation state for both metal centers, corresponding to a d^7^ electronic configuration. The crystallographic data and refinement details are listed in Table 1.

**Table 1:** Crystal Data and Structure Refinement for [Co_2_(μ-L1H)_2_(μ-H_2_O)_2_(H_2_O)_4_]­4NO_3_·2H_2_O

**compound**	1
empirical formula	C_10_H_26_Co_2_N_14_O_20_
formula weight	780.31
crystal system	monoclinic
space group	*P*2_1_/*c*
*a* (Å)	10.3954(3)
*b* (Å)	18.9569(6)
*c* (Å)	7.3054(2)
α (°)	90
β (°)	103.6900(10)
γ (°)	90
*V* (Å^3^)	1398.74(7)
temperature (K)	150(2)
*Z*	2
ρ_calc_ (g cm^–3^)	1.853
μ (mm^–1^)	1.298
*F*(000)	796
crystal size (mm^3^)	0.200 × 0.040 × 0.020
2θ range for data collection (°)	2.285–29.178
index ranges	– 14 < *h* < 14
	– 25 < *k* < 25
	– 10 < *l* < 9
reflections collected	54,523
independent reflections	3723 [*R*_int_ = 0.0357]
final *R*_1_, *wR*_2_ [*I* > 2σ(*I*)]	0.0305, 0.0700
final *R*_1_, *wR*_2_ (all data)	0.0332, 0.0747
data/restraints/parameters	3723/0/252
goodness-of-fit on *F* ^2^	1.133
CCDC number	2440271

**Table 2:** Selected Bond Lengths (Å) for Compound **I** [Co_2_(μ-L1H)_2_(μ-H_2_O)_2_(H_2_O)_4_]­4NO_3_·2H_2_O (*i* = Symmetry Operation [−*x* + 2, −*y* + 1, −*z* + 1])

bond	distance (Å)
Co(1)–O(1)	2.1614(12)
Co(1)–O(2)	2.0439(13)
Co(1)–O(3)	2.0445(14)
Co(1)–N(9i)	2.1199(13)
Co(1)–N(3)	2.1435(14)
Co(1)–O(1i)	2.1780(12)

**Figure 1:**
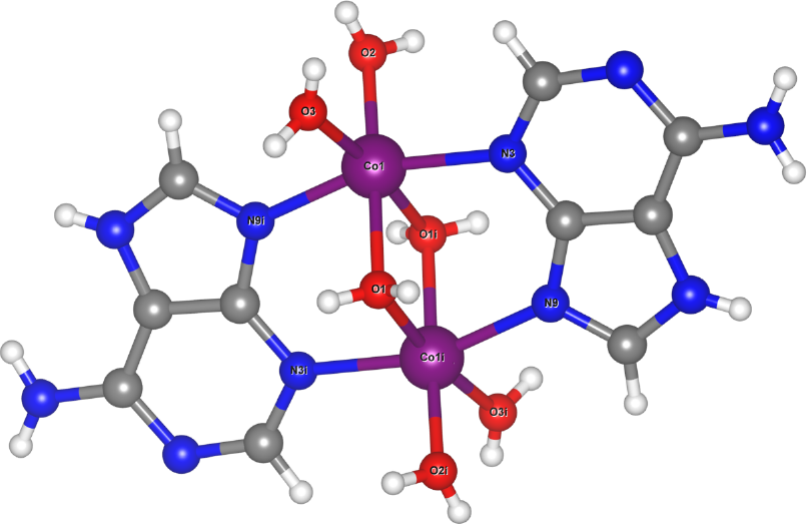
Molecular structure of compound **I** (*i* = symmetry operation [−*x* + 2, −*y* + 1, −*z* + 1]). Nitrate counterions and water molecules were omitted for clarity.

**Figure 2:**
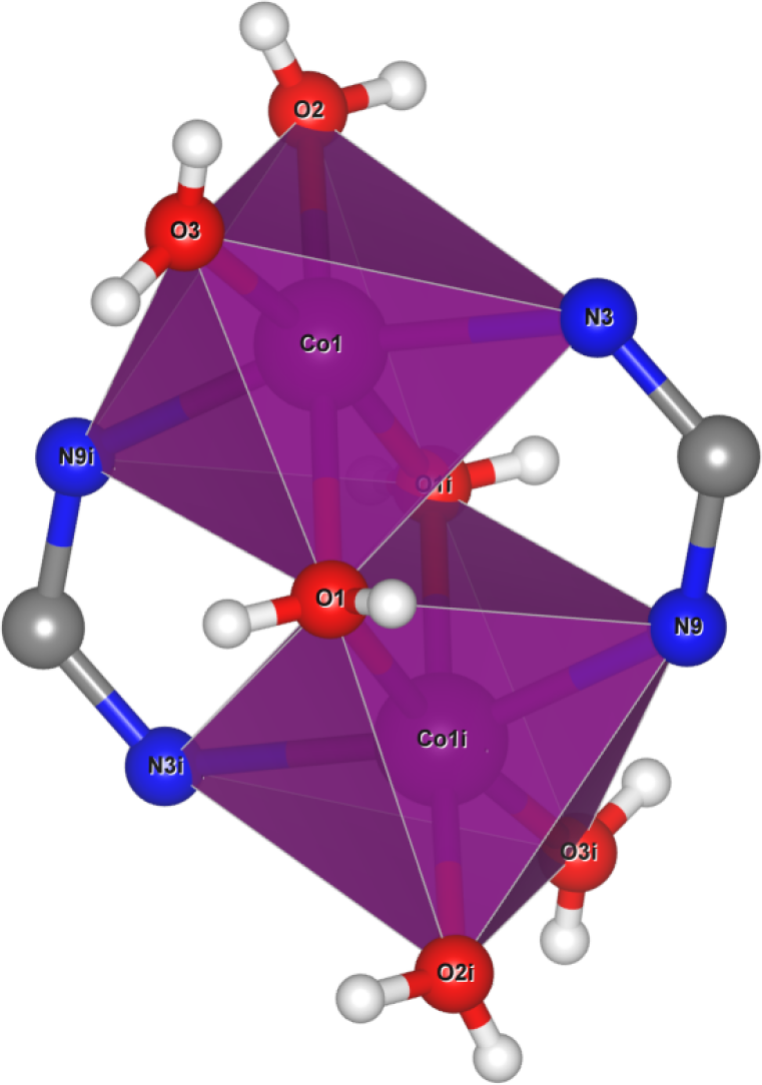
Polyhedral representation of compound **I** (*i* = symmetry operation [−*x* + 2, −*y* + 1, −*z* + 1]). Nitrate counterions, water molecules, and aromatic rings have been omitted for clarity.

In a hexacoordinated ligand field, Co^II^ can adopt either a high-spin state (*S* = 3/2) or, when surrounded by strong-field ligands, a low-spin state (*S* = 1/2). The weak-field character of the bridging and terminal water molecules present here stabilizes the high-spin ^4^ *T*_1*g*_ ground term.^13,14,18^ Strong spin–orbit coupling within this term generates pronounced magnetic anisotropy, reflected in sizable zero-field-splitting parameters *D* and *E*.^19–21^

Axial distortions further split the *t*_2*g*_ manifold, while relativistic effects modulate its separation from the *e*_*g*_ set. Together, these factors govern the effective spin–orbit coupling and, consequently, the temperature-dependent magnetic susceptibility and hysteresis observed for Co^II^ complexes.^20,22,23^

### 2.3 Magnetic Data

Magnetic measurements were conducted using a PPMS platform following the guidelines of Quantum Design systems. The magnetic properties of compound **I** were investigated in a polycrystalline sample through isothermal magnetization curves in the magnetic field range from 0 to 9 T and isofield magnetization in the temperature range of 2–300 K. As the material does not exhibit long-range magnetic order, no special treatment was needed. The isofield magnetization data were subsequently used to calculate the magnetic susceptibility according to χ­(*T*) = *M*(*T*)/*B*, where *M*(*T*) is the magnetization and *B* is the constant applied magnetic field. To quantitatively analyze the magnetic properties, the experimental χ*T* data were fitted using the DAVE-MagProp software.^24^ In this approach, the magnetic susceptibility is calculated as the orientational average, χ = (χ_*x*_ + χ_*y*_ + χ_*z*_)/3, and the goodness of the fit is assessed using Pearson’s chi-squared criterion. The quality of the fit was evaluated through the reduced chi-squared value, yielding χ_red_^2^ = 0.09, which indicates excellent agreement between the experimental data and the model, as shown in the left panel of Figure 3. The same set of parameters obtained from the fit of χ*T* was subsequently used to fit the field-dependent magnetization, as shown in the right panel of Figure 3. As can be seen, the excellent agreement between the susceptibility and magnetization fits using this parameter set strongly validates the robustness of the applied magnetic model.

**Figure 3:**
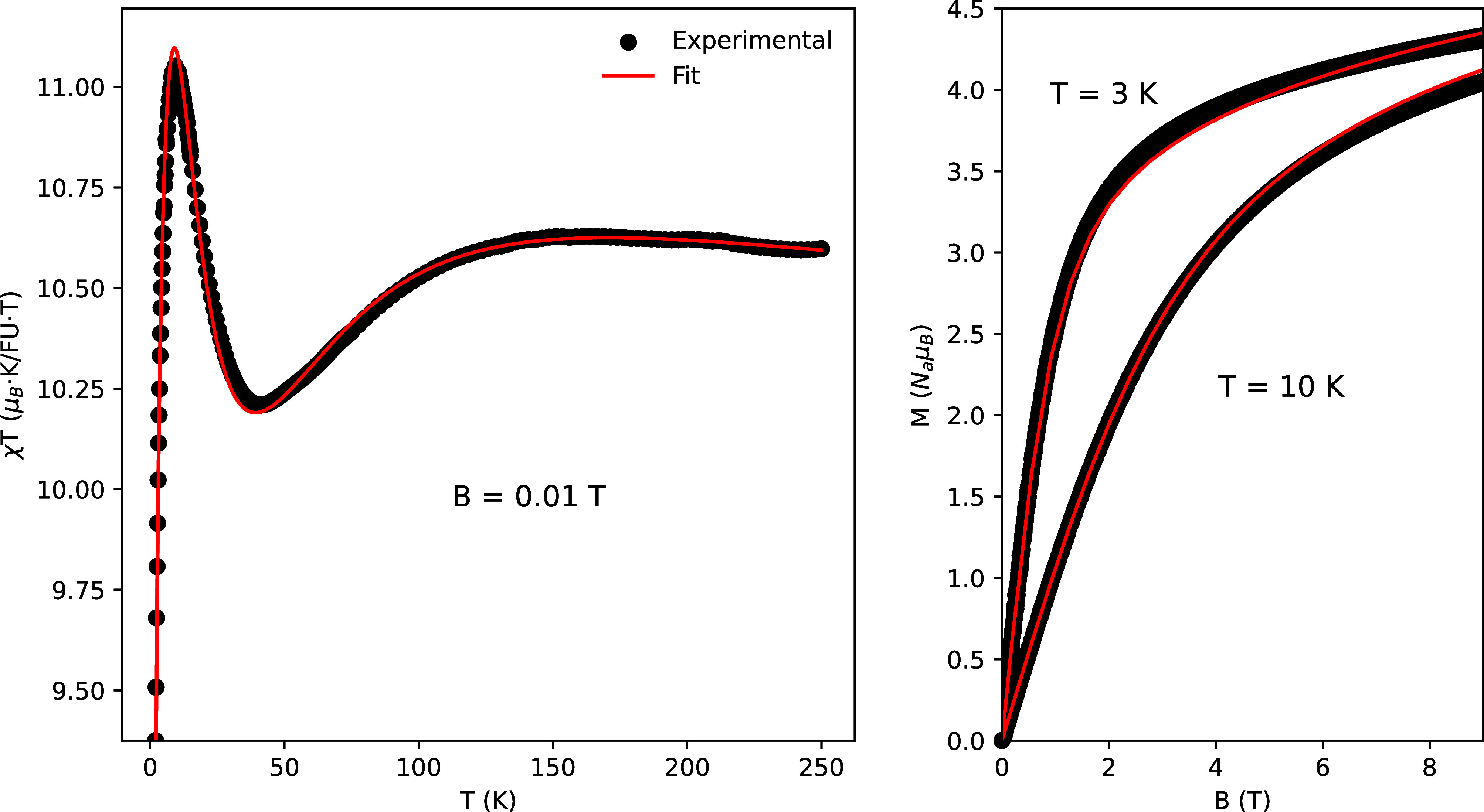
Experimental data (circles) and theoretical fit (solid red line) of χ*T* (left panel) and field dependence of the magnetization (right panel) for [Co_2_(μ-L1H)_2_(μ-H_2_O)_2_(H_2_O)_4_]­4NO_3_·2H_2_O. The strong agreement between the susceptibility and magnetization fits using this parameter set underscores the robustness and reliability of the extracted parameters.

At 3 K, the magnetization approaches saturation at *B* = 9 T, reaching a value of *M* ^exp^ = 4.33 *N*_*A*_μ_*B*_, which is significantly lower than the theoretical saturation limit *M* ^theo^ = *g*_iso_ *S*_max_ = 6 *N*_*A*_μ_*B*_, assuming *g*_iso_ = 2 and *S*_max_ = 3. This reduced saturation magnetization indicates the presence of magnetic anisotropy in addition to an antiferromagnetic exchange interaction between the two Co atoms.

A similar effect is observed in the χ*T* versus temperature curve shown in the left panel of Figure 3. At high temperatures, the magnetic susceptibility follows the Curie law, for which χ*T* = *C* remains constant, where *C* = 2*g* ^2^μ_*B*_^2^ *S*_max_(*S*_max_ + 1)/3*k*_B_. The theoretical Curie constant per dimer is *C* ^theo^ = 6.72 μ_*B*_ ·K/FU·*T*, whereas the experimental value extracted from the χ*T* data is significantly larger, *C* ^exp^ = 10.61 μ_*B*_ ·K/FU·*T*, exceeding the spin-only prediction. This substantial enhancement can be attributed to strong spin–orbit coupling effects, which increase the effective Landé factor, as well as the presence of zero-field splitting. For these reasons, in the following analysis, we will assume that both Co ions remain in the HS state and explicitly include the ZFS anisotropy term in the effective Hamiltonian introduced in the next section.

## 3 Effective Hamiltonian

The crystal structure of the system leads to the inclusion of a Heisenberg exchange interaction term. To account for the anisotropic effects arising from partial quenching of the orbital angular momentum and the coordination environment of Co­(II), the model incorporates axial (*D*) and rhombic (*E*) anisotropy parameters. As both Co­(II) ions are symmetric and exhibit the same hexacoordination, the axial and rhombic parameters are identical for both atoms. These anisotropy parameters significantly influence the magnetic properties of Co­(II) complexes, as they are governed by the crystal field and spin–orbit coupling. Notably, previous studies have demonstrated that pronounced anisotropy in hexacoordinated Co­(II) complexes results from the interplay between spin–orbit coupling and distortions in the coordination geometry.^18^ Interdimer couplings were neglected since the distance between Co dimers is 7.3 Å. Furthermore, no experimental evidence of long-range magnetic ordering was observed, which supports this assumption. Consequently, we propose the effective Hamiltonian given by

$$H=-J{\vec{S}}_{1}\cdot {\vec{S}}_{2}+\sum_{i=1}^{2}\left\{D\left(S_{iz}^{2}-\frac{1}{3}S_{i}^{2}\right)+E(S_{ix}^{2}-S_{iy}^{2})-\mu_{B}\vec{B}\cdot \overset{↔}{g}\cdot {\vec{S}}_{i}\right\}$$ 1

where *S*_*i*_ (*i* = 1, 2) represents the spin operators for both Co­(II) in the high-spin state, $\overset{↔}{g}$ is the diagonal tensor for the Landé factors *g*_*x*_, *g*_*y*_, and *g*_*z*_. These Landé factors are crucial in understanding the magnetic anisotropy of Co­(II) complexes, as highlighted in ref 21. Spectroscopic investigations have demonstrated that the variation of the gyromagnetic ratio *g* along different crystallographic axes can provide valuable insights into electronic distribution and ligand interactions, as discussed in ref 23. Additionally, EPR studies have shown that refined gyromagnetic ratios can be used to establish correlations between magnetic anisotropy and zero-field splitting parameters, as reported in ref 22.

This effective Hamiltonian was used to calculate the average powder χT as a function of temperature, with the model parameters optimized to fit the experimental data (see the left panel of Figure 3). Additionally, diamagnetic contributions (χ_D_ = −1.8 · 10^–4^ μ_*B*_/FU · *T*) from the system were also included. The same model was also employed to fit the magnetization as a function of the applied magnetic field, as shown in the top panel of Figure 3. The resulting fitted parameters are given in Table 3.

**Table 3:** Fitted Parameters Obtained from the Magnetic Susceptibility-Temperature (χ*T*) Data for Compound **I**

compound	*J* (K)	*D* (K)	*E* (K)	*g*_*x*_	*g*_*y*_	*g*_*z*_
**I**	–8.6(8)	89(5)	23(2)	2.5(1)	2.5(1)	2.4(1)

## 4 Results and Discussion

At first glance, the exchange constant *J* = −8.6 K indicates antiferromagnetic coupling between the cobalt ions, consistent with the reduction of χ*T* as the temperature approaches the exchange energy scale. This trend is clearly observed in the left panel of Figure 3, where χ*T* decreases as *T* → 0, reflecting the stabilization of a singlet ground state and the consequent suppression of the magnetic susceptibility. Hexacoordinated high-spin Co­(II) dimers can exhibit either antiferromagnetic or ferromagnetic coupling behavior, depending on how the bridging occurs between the metal centers, as the magnetic interaction is usually governed by superexchange pathways. Numerous examples can be found in the literature, for instance, both [Co_2_(μ-OAc)_2_(μ-AA)­(urea)­(tmen)_2_]­[OTf] and [Co_2_(μ-H_2_O)­(μ-OAc)_2_(OAc)_2_(tmen)_2_] exhibit antiferromagnetic coupling, with *J* = −5.18 K and *J* = −1.01 K, respectively, whereas [Co_2_(μ-OAc)_3_(urea)­(tmen)_2_]­[OTf] shows ferromagnetic coupling with *J* = 25.89 K^27^ due to the substitution of the hydroxamate.

Furthermore, the obtained axial and rhombic zero-field splitting parameters are *D* = 89 K and *E* = 23 K, respectively, which are characteristic of systems where the electronic states experience significant crystal field splitting. This magnetic anisotropy is also reflected in the Landé factors, with fitted values *g*_*x*_ = *g*_*y*_ = 2.5 and *g*_*z*_ = 2.4, indicating that the *xy* plane is the easy plane of magnetization, consistent with the positive value of D and the condition *g*_*x*_, *g*_*y*_ > *g*_*z*_. Although different effective Hamiltonians are commonly employed in the literature, this anisotropic behavior is frequently observed in Co­(II) dimers, where D is typically positive and *g*_*x*_, *g*_*y*_ > *g*_*z*_, for instance, the six dinuclear Co­(II) complexes^25^ modeled by a general bilinear exchange shows large values of D ranging from 71.9 to 143.8 K, where all the complexes are antiferromagnetic with easy-plane magnetization. Representative examples include [Co_2_(3-fuc)_4_(isonia)_4_] and [Co_2_(2-fuc)_4_(isonia)_4_],^26^ which were modeled using a similar Hamiltonian without the rhombic term. For these compounds, the reported parameters are *g*_*x*_ = *g*_*y*_ = 2.527, *g*_*z*_ = 2, and *D* = 90.21 *K* for the first complex, and *g*_*x*_ = *g*_*y*_ = 2.5, *g*_*z*_ = 2, and *D* = 52.34 K for the second one, which also shows an easy-plane behavior and antiferromagnetic coupling. The parameters of these complexes can be seen in Table 4.

**Table 4:** Magnetic Exchange and Anisotropy Parameters for Several Hexacoordinated Dinuclear Co­(II) Complexes^a^

compound	*J* (K)	*D* (K)	*g*_*x*_	*g*_*y*_	*g*_*z*_
[Co_2_(μ-L1H)_2_(μ-H_2_O)_2_(H_2_O)_4_]4NO_3_·2H_2_O	–8.6	89	2.5	2.5	2.4
[Co_2_(3-fuc)_4_(isonia)_4_]^a^	–2.53	90.21	2.527	2.527	2
[Co_2_(2-fuc)_4_(isonia)_4_]^a^	–3.44	52.34	2.5	2.5	2
[Co_2_(H_2_O)(PhCO_2_)_4_(py)_4_]·0.5(PhCO_2_H)·1.5(MePh)^b^	–1.57	132.9	2.52	2.52	2.17
[Co_2_(H_2_O)(PhCO_2_)_4_(Mepy)_4_]^b^	–1.01	72.9	2.31	2.31	2.01
[Co_2_(H_2_O)(PhCO_2_)_4_(iqu)_4_]·iqu^b^	–3.49	143.3	2.54	2.54	2.00
[Co_2_(H_2_O)(PhCO_2_)_4_(fupy)_4_]^b^	–1.29	98.8	2.54	2.54	2.00
[Co_2_(H_2_O)(PhCO_2_)_4_(Mefupy)_4_]^b^	–2.34	114.9	2.70	2.70	2.59
[Co_2_(H_2_O)(PhCO_2_)_4_(Me_2_fupy)_4_]^b^	–1.61	111.5	2.74	2.74	2.54

a Note: Superscripts ^*a*^ and ^*b*^ refer to values taken from refs 25 and 26, respectively. It is worth noting that the analysis in the superscript “a” does not include the rhombic anisotropy parameter *E*. In contrast, the the analysis in the superscript “b” adopts a more general description of the magnetic behavior, incorporating nondiagonal components of the *D*-tensor, which also does not include the rhombic term.

## 5 Conclusions

In summary, the present study offers a quantitative magnetic characterization of a hexacoordinated cobalt­(II) dimer with emphasis on its anisotropy and exchange coupling, as determined from a consistent analysis of susceptibility and magnetization data. The dinuclear Co­(II) molecular magnet [Co_2_(μ-L1H)_2_(μ-H_2_O)_2_(H_2_O)_4_]·4NO_3_·2H_2_O, which has been structurally characterized, was successfully subjected to the magnetochemical analysis, where both hexacoordinated Co­(II) centers are antiferromagnetically coupled (*J* = −8.6 K) and exhibit pronounced axial (*D* = 89 K) and rhombic anisotropy (*E* = 23 K), consistent with previous studied metal complexes in the literature. This anisotropy was also captured in the parameters of the anisotropic Landé factors, where *g*_*x*_ = *g*_*y*_ = 2.5 and *g*_*z*_ = 2.4, showing easy-plane magnetization, for which can be further corroborated by high-frequency EPR. In addition, it is worth highlighting that the consistency between the fits of the susceptibility and magnetization data with the set of parameters obtained supports the reliability of the applied magnetic model. Therefore, by presenting a new example of a dinuclear Co­(II) complex with comprehensively characterized magnetic anisotropy, this work furnishes additional experimental data that advances the fundamental understanding of spin–orbit coupling phenomena in molecular magnetic systems.
